# The Results of Radium Therapy

**Published:** 1914-03-21

**Authors:** 


### The Results of Radium Therapy.

One of the striking features of the collection of expert views upon the facts of radio-therapy, made for the purpose of this article, is the wide divergence of opinion as to its effects in different diseases. There is, however, one principle upon which all seem to be agreed, and, as it is of vast importance, it will be well to emphasise it at once.

*The results of the radium treatment of malignant diseases do not entitle it to displace operative surgical treatment as the mainstay of the defence against those diseases.* That is to say, operation holds the field still as by far the best hope the patient has, whenever such an operation can be planned with a reasonable chance of eradicating all the foci of the disease. For inoperable cases, where the situation or the extent of the disease make a radical operation impossible, then radio-therapy often reduces suffering and often prolongs life; sometimes it even saves it. The recognition of these truths is vital to the future progress of radium therapy. It is the fact that astonishingly good results have been secured in many cases of malignant disease which appeared hopeless. It is true that a percentage of such patients have been restored for a time to fairly good health, and that no other known agency could have accomplished so much. It *may even turn out to be true* that real, complete, permanent cure of malignant disease in such cases does now and then occur. But this is uncertain as yet, and it will in the nature of things be some few years before a definite pronouncement to this effect can be legitimately made; whereas it is an indisputable and well-established fact that operative treatment results in a fair proportion of cases in genuine permanent cure; that this percentage is steadily rising with the greater perfection of modern surgical methods; and that for some especially favourable sites it amounts to 50 per cent. of cases, or even more. It is, therefore, of the highest importance that, until radio-therapy can show results a vast deal better than any so far obtained, no malignant tumour which is operable shall be treated without operation. Radium therapy or $x$-ray therapy may be useful as an adjunct of, but *not as a substitute for surgery.*

So far the ground is safe. But attempts to generalise further than this are extremely hazardous. By considering *seriatim* the various diseases for which radio-therapy is most employed, the conflicting views of experts, its uses, abuses, and limitations may be set forth. Necessarily all conclusions are tentative, and liable to reversal as experience and scientific knowledge accumulate. In this connection the recent debate at the Berlin Medical Society, to which reference has already been made, is of interest. The first speaker, a man of world-wide eminence, said he could get curative effects (upon tumours) to a depth of 4 cm. (about one inch and three-fifths), but no further. He expressed the belief that deep-acting $x$-rays are *at least* of value equal to radium. The next speaker emphasised the danger of small doses of radium in accelerating the rate of growth of tumours. Another thought that radium is of use for surface growths, but that deep-seated disease is not benefited. Another, who gives enormous doses, reported results so favourable that they seem to have excited incredulity. Finally, another surgeon, whose name is known throughout the surgical world, denied any merit in radio-therapy at all. This brief recital will give some conception of the chaotic state in which expert opinion finds itself just now; it shows the great need for caution, and the immorality of arousing by unjustifiable dogmatisation premature expectations among the lay public.

There are three methods at least in which radium and operative treatment may be combined. Thus sometimes an inoperable mass may so shrink under the influence of radium that it becomes operable. This is rare, and some experienced workers have yet to come across such a case; others speak of it as of only moderate rarity. The truth probably is that such an event does sometimes occur, but very seldom. Then, again, sometimes the removal by a surgeon of a big mass of malignant disease affords the radium expert a chance of applying radiations to that which remains with a far better chance of success than if nothing had been removed. And, thirdly, the prophylactic radiation of parts from which a surgeon believes he has completely removed a growth offers a field for radium treatment the limitations of which are not yet known. As far as $x$-rays are concerned, this prophylactic radiation method has not answered expectations. Whether a better report will ultimately be given about radium remains to be seen. With these premonitory warnings, we turn to the discussion in detail of the morbid states for which radio-activity has been most employed as a therapeutic agent.

### Malignant Diseases.

Radiations, whether produced by radium or by any other source of radio-activity, have been used in the treatment of a great variety of diseases during the past decade. For a variety of reasons that side of radio-therapy which has most caught public attention is its use for the various kinds of malignant disease. It may be well, therefore, to discuss this first, before turning to those other conditions of inflammation or of innocent new growth which respond more or less satisfactorily to radiations.

Amongst the mass of literature which has already accumulated on the radium treatment of malignant disease, it is not easy to pick one's way and to get down to first principles. It appears, however, to be certain that some types of malignant growth, as, for instance, round-cell sarcoma, are destroyed or damaged much more easily than others, such as epithelioma. It is also true that malignant growths in some situations, such as the cervix of the uterus, are more amenable to treatment than growths in some other situations, such as the pancreas. But when any attempt is made to summarise the effects of radium on malignant growths more dogmatically and categorically than this, the wide differences of opinion which prevail among the experts entirely prohibit the attempt.

### Rodent Ulcer.

This very chronic form of skin cancer, usually of exceedingly slight malignancy compared with most other forms, does very often react satisfactorily to radium. Some authorities even speak of radium as a certain cure for cases in an early stage and as giving a fair chance of cure in later stages. In point of fact the slow progress of rodent ulcer, even when left entirely untreated, and the whole natural history of the disease with its spontaneous fluctuations and intermissions, make it very unsafe to pronounce so optimistically as this just at present. It is, however, noteworthy that several cases have been reported in which a rodent ulcer, after having healed as a consequence of exposure to radium, has later on started afresh in a much more acute and intractable form. It is also to be noted that some cases of rodent ulcer do not seem to be benefited at all by radium, but react speedily to $x$-ray treatment: and, conversely, when $x$-rays have proved disappointing radium sometimes acts like a charm. One leading authority distinguishes, as a result of a large experience, two types of the disease. The ulcerative type he finds very refractory to radium; but the other type, characterised by excess of growth rather than of ulcer, does extremely well. Whether this clinical classification explains the contradictory reports on the reaction of rodent ulcer to radium remains to be seen. The practical point seems to be this: that for early cases, where a surgical operation can be planned to remove the whole growth with a margin of healthy tissue, it should unquestionably be done. For more advanced cases both radium and $x$-rays should be tried, in the hope that one or other will do good. The chance of a permanent cure in such cases is slight, but not altogether negligible.

### Some Alimentary Cancers.

By common consent cancer of the tongue is not often favourably influenced by radium, though rather more encouraging results are reported lately from the use of very small tubes containing emanation. Good authorities have even suggested that it is not justifiable to treat cancer of the tongue with radium in view of the badness of the results. Certainly operative measures, though they are not so hopeful as in most other regions, have never called forth such expressions of despair as this. Epitheliomata of the pharynx, larynx, œsophagus, tonsil, naso-pharynx are apparently somewhat less refractory to radium. A few very striking cures of what seemed to be quite hopeless growths in these situations have been reported. Caution in accepting such cases as real cures is necessary, as the benefit, though often great, frequently turns out to be merely temporary. Cancer of the lip does better; but is, of course, far more frequently operable, and with excellent results. It would be disastrous in the extreme to abandon operative removal of such growths in favour of radium treatment, though the latter is worth a trial in advanced and in operable cases. In all these cancers the glands of the neck and mediastinum are liable to be involved by metastases. The chance of curing these by radium is very slight, but

(Continued on p. 679.)

### THE RESULTS OF RADIUM THERAPY.

(*Continued from p. 674.*)

marked improvement for a time is not very uncommon. Indeed, the failure of radium to prevent metastases, even when it abolishes the primary growth entirely, is one of the most disappointing features of its application to malignant disease.

About cancer of the rectum the reports are conflicting. Some claim that the annular types do well, but the plaques of projecting growth badly; others take just the opposite view. Cancer of the stomach and intestine are naturally less amenable because of their inaccessibility, and the reports of treatment by radium have not so far been encouraging.

### Cancer in Other Regions.

Cancer of the breast, by reason of its extreme frequency and of its position, has been treated with radium in a very large number of cases. It is especially here that the prophylactic radiation after a radical operation has been employed, with results which time has yet to disclose. Even in cases so advanced as to appear hopeless there have been occasionally very remarkable results. Unfortunately there have been also a great many failures, and, even when success with the growth itself has been striking, metastases seem to be just as common as before. It has been suggested that to prevent these *x*-ray treatment on a grand scale should be employed. Paget's disease of the nipple can, it is said, usually be made to disappear by radium; but once again the tendency to metastases is not obviated, and the treatment is, therefore, unsatisfactory.

Cancer of the cervix of the uterus is so frequently inoperable when the diagnosis is first made that radium has been much employed. On the whole, the success attained has been promising. Some have actually advocated the use of radium with diathermic treatment in preference to surgical operation in operable cases; but this advanced position is probably unjustifiable as yet, though the results even of Wertheim's radical operation for this disease are not by any means as good as could be wished. Cancer of the body of the uterus is, of course, more favourable for surgical removal: it is said to have yielded some good results in inoperable cases when treated with radium. On the whole, the uterus would seem to be a site where radium treatment of cancer may quite possibly have a brilliant future in store.

Of cancer of the prostate as much cannot be said. Improvement has been reported in a few cases, but cure seems to be still improbable. Incidentally, the radium treatment of simple enlargement of the prostate (adenoma) is much more likely to make headway. Vesical growths, whether villous, papillomatous, or flat ulcers, are often improved by radium; but in the nature of things cure is hardly to be expected, except in rare cases by surgical operation.

Melanotic sarcoma, wherever situated, is an exception to the general rule that sarcomata do well. Lymphosarcoma, on the other hand, reacts uncommonly well to radium treatment: there is a consensus of opinion to this effect, and it is also said that lymphadenoma does even better. This latter disease is so extensively disseminated as a rule by the time it is diagnosed that the chance of curing it by any treatment must always be remote. It is true that some authors suggest that the apparently favourable results of radium for sarcoma are due to the difficulties of diagnosis (even when a microscopical examination has been made), and that some of the reported cures have in reality been cases of inflammation and not of growth at all.

It will be realised that one of the most serious limitations of radium in the treatment of malignant disease is its inability to prevent metastases. Indeed, at the meeting of the Medical Society of London, to which reference has been already made, one of the speakers laid down as a condition of calling any agent a "cure" for cancer, that it should be capable of causing the disappearance not only of the primary growth but of all secondary glandular and metastatic deposits also. It is needless to say that no such valuable remedy is in sight at present. Certainly radium has no such power; for one of the striking features of the reports of cancer cases in which radium has caused the original tumour to disappear is the frequency with which ultimate secondary deposits appear and cause death. There seems to be some ground also for the belief that, as with *x*-rays, the first exposure to radium is often the most beneficial, and that afterwards each successive treatment does less and less good until there is no effect at all.

### Effects of Radium on Innocent Tumours.

Whether there really is a selective action of radium radiations upon the cells of malignant growths is not yet established. If there is, it is at least conceivable that the same may hold good of innocent tumours; for the cells of these, though not so alien as those of cancer, are at any rate interlopers amidst the normal structure of the tissues. So far this question has not attracted much notice. But, in spite of this comparative neglect of the theoretical basis of radium treatment, many innocent tumours have been empirically subjected to radiation, with a fair proportion of satisfactory results.

Fibroids of the uterus have been the subject of many trials along these lines. These tumours, though clinically innocent, are so exceedingly common, so distressing in many cases, and affect the general health so greatly, that the success of any non-operative form of treatment would be a great blessing to womankind. For years *x*-rays have been experimented with; but, although gynæcologists here and there have reported enthusiastically, there is no doubt that the treatment is a failure in all but a very small percentage of cases. The reports upon radium treatment vary very greatly. Some are highly optimistic, but it is noteworthy that many of these emanate from men who are equally sure that *x*-rays will cure this disease. Others are much more cautious; but there seems to be unanimity that improvement, at least, is secured in a fair proportion of cases. At the Medical Society's discussion last week the opinion was expressed that for most cases operative treatment is still greatly preferable; but that menorrhagia and metrorrhagia can be controlled by radium. In other words, this particular symptom can be relieved; and if it is the only one that causes complaint, and the growths are in themselves small, the radiation treatment is justifiable. A tube of 100 mg. is placed in the vagina, actually in the cervix if possible; and an equal dose on the abdominal wall. For four or five weeks no improvement may be expected, but after that there is a steady diminution of the flux. It has been suggested also that the abdominal radiations should be applied in two parts, one on each side of the middle line. The object of this is to avoid the risk of burns on that part of the skin through which an incision may afterwards be required, if operative treatment becomes necessary. This caution in itself demonstrates the fact that radium is not always successful.

### Exophthalmic Goitre.

This refractory and serious disease has long been a stumbling-block to the medical profession. Medical treatments, of which there are scores of varieties, succeed in a fair proportion of cases; physicians think in a large proportion. Surgery also succeeds sometimes, though the operation is peculiarly dangerous: surgeons believe that their successes are numerous enough to discount the admitted risk. *X*-rays have seemed to cure obstinate cases in which all other remedies have failed; but, like the preceding forms of treatment, cannot be relied upon. Even when *x*-rays succeed, the innate tendency of the disease to recur is still to be reckoned with. Much the same is true of radium. Some really wonderful successes have been reported, but failures are also common. Whether the successes will be permanent remains to be proved. At least radium (and *x*-rays) are worth a trial, if medical treatment fails, before resorting to surgery.

### Birth Marks.

Since nævi are in most cases situated on an exposed surface, usually the face, any method of treatment which will leave no scarring will be preferred to surgical operation. The latter, in addition, is only feasible for quite small marks. A few years ago electrolysis had a great vogue, but is seldom used now. Then *x*-rays were advocated, and some cases did well when thus treated. Four or five years ago carbon dioxide snow was introduced, and gives results which are distinctly satisfactory. There are, however, cases, such as "port-wine marks" and cavernous nævi, which are not suitable for the snow treatment; and for these radium is sometimes beneficial. Port-wine stain, unfortunately, does not do so well as some of the other varieties of angioma. In all these cases great care and experience are necessary to avoid the formation of unsightly telangiectases on the site of the nævus. This is a question of dosage and filtration too technical to be discussed here; nor, indeed, do the experts all agree about it.

### Other Skin Diseases.

For keloid there is a consensus of opinion that radium acts like a charm. This is all to the good, since no other remedy has any effect upon this obstinate condition. Lupus erythematosus can be improved, but hardly ever cured, by radium: here, again, a particularly intractable lesion is in question. Of psoriasis many favourable reports have been published; but the best authorities are convinced that relapse is the rule. Lichen, chronic eczema, molluscum contagiosum, and sebaceous growths have all been treated successfully by radium. Of lupus conflicting accounts are given. Some workers are very disappointed with the results of radium treatment; but others have criticised the details as to screening and dosage, and are much encouraged by their own cases. Probably the less optimistic views are the more justifiable at present. Operative surgery and Finsen light are preferable to radium for lupus in the opinion of undoubted experts in radio-therapy.

Warts, corns, and papillomata react very well to radium. Most of these troublesome innocent growths can be effectively and easily cured by surgical means of course. But there are some situations, as, for instance, the larynx, where non-operative measures are most valuable; and there are, in any case, those individuals to reckon with who have an abhorrence of "the knife." Gonorrhœal warts, an especially troublesome variety, are also said to be very successfully treated by radium. Moles can also be got rid of in the same way, but less easily than warts; and scarring follows. If the views of those who regard every mole as a potential site of melanotic sarcoma, to be rooted out forthwith, gain universal adoption, radium will doubtless be a good deal used in the process of extermination.

A good many sensational reports of cures of deafness by radium and by *x*-rays have been in circulation this last year or two. It seems to be the unanimous opinion of otologists that these reports have no substantial foundation. Both radium and ultra *x*-rays (which are nearer in character to the $\gamma$-rays of radium than any other form of *x*-rays) are authoritatively described as total failures by experts in this specialty.

Some little time ago much interest was aroused by favourable reports from the Radium Institute of the treatment of rheumatoid arthritis by imbibition of emanation-water. These reports were received with some scepticism, which seems to have been partly justified. At present it is merely claimed that some cases do remarkably well. Those in whom good results are most to be expected are persons under forty, with multi-articular lesions and peri-articular type. Chronic gout has also been treated in the same way, and good results have been published. Emanation-water has also been recommended for anæmia, diabetes, and cancer; but great caution is necessary in accepting all these suggestions.

